# Identification of microtubule-associated biomarkers in diffuse large B-cell lymphoma and prognosis prediction

**DOI:** 10.3389/fgene.2022.1092678

**Published:** 2023-01-24

**Authors:** Wenqi Wu, Su Liu, Linyan Tian, Cheng Li, Yanan Jiang, Jinhuan Wang, Yangyang Lv, Jing Guo, Donghui Xing, Yixin Zhai, Huimeng Sun, Yuhang Li, Luying Zhang, Xiang He, Kaiping Luo, Hongjie Zhan, Zhigang Zhao

**Affiliations:** ^1^ Department of Hematology, Tianjin Medical University Cancer Institute and Hospital, National Clinical Research Center for Cancer, Key Laboratory of Cancer Prevention and Therapy, Tianjin’s Clinical Research Center for Cancer, Tianjin, China; ^2^ Department of Medical Oncology, Tianjin First Central Hospital, School of Medicine. Nankai University, Tianjin, China; ^3^ Department of Pharmacy, Shandong Cancer Hospital and Institute, Shandong First Medical University and Shandong Academy of Medical Sciences, Jinan, China; ^4^ Department of Gastroenterology, Tianjin Medical University Cancer Institute and Hospital, National Clinical Research Center for Cancer, Key Laboratory of Cancer Prevention and Therapy, Tianjin’s Clinical Research Center for Cancer, Tianjin, China

**Keywords:** DLBCL, microtube-associated genes, prognostic model, targeting therapy, *TMEM63A*

## Abstract

**Background:** Diffuse large B-cell lymphoma (DLBCL) is a genetically heterogeneous disease with a complicated prognosis. Even though various prognostic evaluations have been applied currently, they usually only use the clinical factors that overlook the molecular underlying DLBCL progression. Therefore, more accurate prognostic assessment needs further exploration. In the present study, we constructed a novel prognostic model based on microtubule associated genes (MAGs).

**Methods:** A total of 33 normal controls and 1360 DLBCL samples containing gene-expression from the Gene Expression Omnibus (GEO) database were included. Subsequently, the univariate Cox, the least absolute shrinkage and selection operator (LASSO), and multivariate Cox regression analysis were used to select the best prognosis related genes into the MAGs model. To validate the model, Kaplan-Meier curve, and nomogram were analyzed.

**Results:** A risk score model based on fourteen candidate MAGs (*CCDC78, CD300LG, CTAG2, DYNLL2, MAPKAPK2, MREG, NME8, PGK2, RALBP1, SIGLEC1, SLC1A1, SLC39A12, TMEM63A, and WRAP73*) was established. The K-M curve presented that the high-risk patients had a significantly inferior overall survival (OS) time compared to low-risk patients in training and validation datasets. Furthermore, knocking-out *TMEM63A*, a key gene belonging to the MAGs model, inhibited cell proliferation noticeably.

**Conclusion:** The novel MAGs prognostic model has a well predictive capability, which may as a supplement for the current assessments. Furthermore, candidate TMEM63A gene has therapeutic target potentially in DLBCL.

## 1 Introduction

Diffuse large B-cell lymphoma (DLBCL) is the most common lymphoid neoplasm, has invasive behavior and a complex origin, and is heterogeneous in clinical presentation, immunophenotype, and molecular genetics (Locke et al., 2017; Kimani et al., 2021; Jiang et al., 2022). Although combination chemotherapy cyclophosphamide, doxorubicin, vincristine, and prednisone (CHOP) containing rituximab serves as the backbone of treatment, approximately 30%–40% of patients experience treatment failure or an inevitable relapse, and the number of DLBCL-related fatalities continues to increase (Maurer et al., 2014; Miao et al., 2019; Matasar et al., 2021). Conventional prognostic evaluation methods, such as the International Prognostic Index (IPI) score and 2-deoxy-2-[F-18]-fluoro-D-glucose (FDG)-PET/CT scan are insufficient to elucidate the clinical diversity of DLBCL (Zwezerijnen et al., 2021). In addition, BCL2 and TP53 mutations are considered prognostic indicators in DLBCL patients (Qin et al., 2021). Nevertheless, the effectiveness of these identified molecular markers has been limited. Therefore, predicting the survival rate of patients with a heterogeneous malignancy such as DLBCL remains challenging (Meyer et al., 2011).

Exploration of the use of microtubules has shown potential positive effects in prognosis prediction in cancer patients. As a major part of the eukaryotic cytoskeleton, microtubules serve as molecular highways and contribute to the exchange of cellular cargo (Roehlecke and Schmidt, 2020; Liang et al., 2022). Furthermore, they consist of spindle apparatus that play a crucial role in the correct attachment and segregation of chromosomes during cell division (Kavallaris, 2010). Tumor cell invasion has proven to be highly dependent on microtubule cytoskeleton systems, and tubulin is the biochemical target for several clinical anticancer drugs, including vinca alkaloids as microtubule destabilizers ;and paclitaxel as microtubule stabilizers (Cao et al., 2018).

Furthermore, tumor microtubules contribute to the resistance against standard treatment modalities in several solid tumors, where most of the surviving cells are tumor microtubule-connected cells (Osswald et al., 2015; Weil et al., 2017). The newly approved antibody–drug conjugate polatuzumab vedotin for the treatment of relapsed or refractory DLBCL performs an essential role *via* prevention of tubulin polymerization. However, the association between microtubules, the prognosis of DLBCL, and the potential involvement of microtubule-related genes has yet to be explored.

Considering current studies, we established a microtubule-associated gene (MAG) prognosis prediction model based on mRNA expression using clinical data from DLBCL patients that were accessible from the National Center for Biotechnology Information Gene Expression Omnibus (NCBI GEO). Moreover, the results have indicated promise for the development of targeted interventions against DLBCL.

## 2 Materials and methods

### 2.1 Data source

Clinical data and gene expression profiling data were acquired from the NCBI GEO database. Data series in GSE10846, GSE11318, GSE31312, GSE87371, and GSE56315 were downloaded in a normalized expression matrix file format for retrospective analysis. As shown in Table 1, 33 normal controls and 1360 DLBCL samples were included in our study.

**TABLE 1:** Clinical information of the patients in training and validating datasets.

Cohort	GSE10846	GSE11318	GSE31312	GSE87371	GSE56315
Number of patient Normal/DLBCL	0/414	0/200	470	0/221	33/55
Age (y)	62.5 (14−92)	64 (14−88)	63 (18−92)	60 (19−87)	NA
Gender Male/Female/NA	172/224/18	110/90	271/199	116/105	NA
GCB/non-GCB/NA	163/232/19	70/100/30	227/243	84/117/20	NA
IPI 0-2/3-5/NA	216/89/109	101/41/58	274/150/46	119/102	NA
Status Alive/Death	249/165	88/112	300/170	168/53	NA

### 2.2 Selection of optimal prognostic genes related to OS

We performed univariate Cox regression analysis to investigate relationships between gene expression and prognostic values. A total of 1474 MAGs (Supplementary Table S1) were retrieved and 596 genes with *p*-values <.05 were retained (Supplementary Table S2). We then performed Lasso penalized Cox regression analysis with 596 microtubule-associated genes. Next, we constructed the multivariate Cox regression analysis using the 53 genes obtained from the Lasso analysis (Supplementary Table S3). Finally, the 14 MAGs that were significantly related to overall survival (OS) in the datasets were extracted (Supplementary Table S4).

### 2.3 Microtubule-associated risk model development and validation

We built a MAG model based on the GSE10846 dataset and validated the predictive capacity of the model using the GSE11318, GSE31312, and GSE87371 datasets.

We calculated the characteristic risk score for every patient using the following formula: risk score = ∑ βi * X. Next, we set up a proper cut-off value and divided patients into low-risk and high-risk groups. Kaplan–Meier (K-M) survival analysis and log-rank test were used to evaluate OS in different groups. The area under the curve (AUC) of the receiver operating characteristic (ROC) curve illustrated the performance of the prognostic signature. The Cox regression model method in the “survival” R package was used for univariate and multivariate analyses to explore the independent prognostic role of the gene signature.

### 2.4 Nomogram construction

A nomogram, including risk score and clinical features (age, gender, and IPI components), was established to predict OS of DLBCL patients at 1, 3, and 5 years. The distinguishing capacity of the nomogram was assessed *via* calibration mapping.

### 2.5 Functional enrichment analysis and immunohistochemical staining

We used the ‘LIMMA’ R package to identify genes differentially expressed between the high-risk and low-risk groups (Supplementary Table S5). Gene Ontology (GO) analysis focused on the upregulated pathways in the high-risk group (Supplementary Table S6). Gene set enrichment analysis (GSEA) was used to find pathway enrichment associated with the differentially expressed genes. Immunohistochemical staining of RALBP1 was downloaded from the HPA database (https://www.proteinatlas.org).

### 2.6 Knockout of the key prognostic gene *TMEM63A*

We used CRISPR-Cas9-mediated sgRNA to target and knockout the *TMEM63A* gene. We then produced a lentivirus with the sgRNA to infect the DLBCL cell lines OCI-LY7 and DOHH2. Next, EdU staining was performed to analyze cell proliferation. TMEM63A-sg1: CAC​CGT​ACT​CAC​TGC​AGA​CGG​AAG​A, TMEM63A-sg2: CAC​CGC​GAT​GAC​AAT​CTC​TGA​AAT​C, and non-targeted: ACG​GAG​GCT​AAG​CGT​CGC​AA.

### 2.7 Statistical analysis

Statistical analyses were carried out, and graphs were generated using GraphPad Prism 8.0 and R.4.1.1 software; *p*-values <.05 indicated significant differences. Expression of MAG mRNA was compared between normal cells and cells from DLBCL patients using the unpaired *t*-test (Figure 2D). The boxplots of risk score (Figures 3C–H) and predicted drug sensitivity (Figures 9A–I) were analyzed using the Wilcoxon test. The K-M curves were analyzed using the log-rank test. The bar plots of cell proliferation were compared using the unpaired *t*-test.

## 3 Results

### 3.1 Construction of the predictive signature model

**FIGURE 1:**
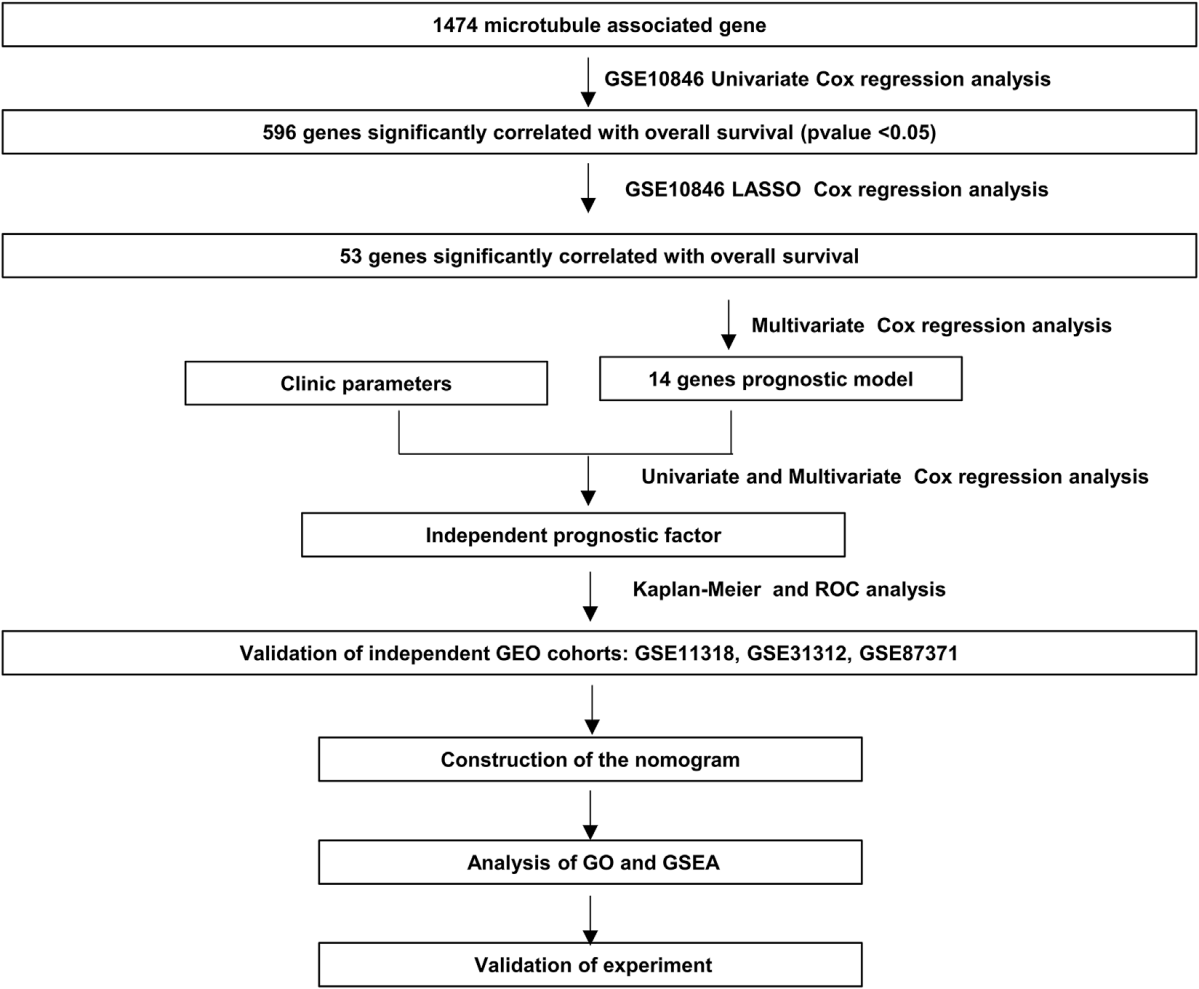
Processes of the study.

**FIGURE 2:**
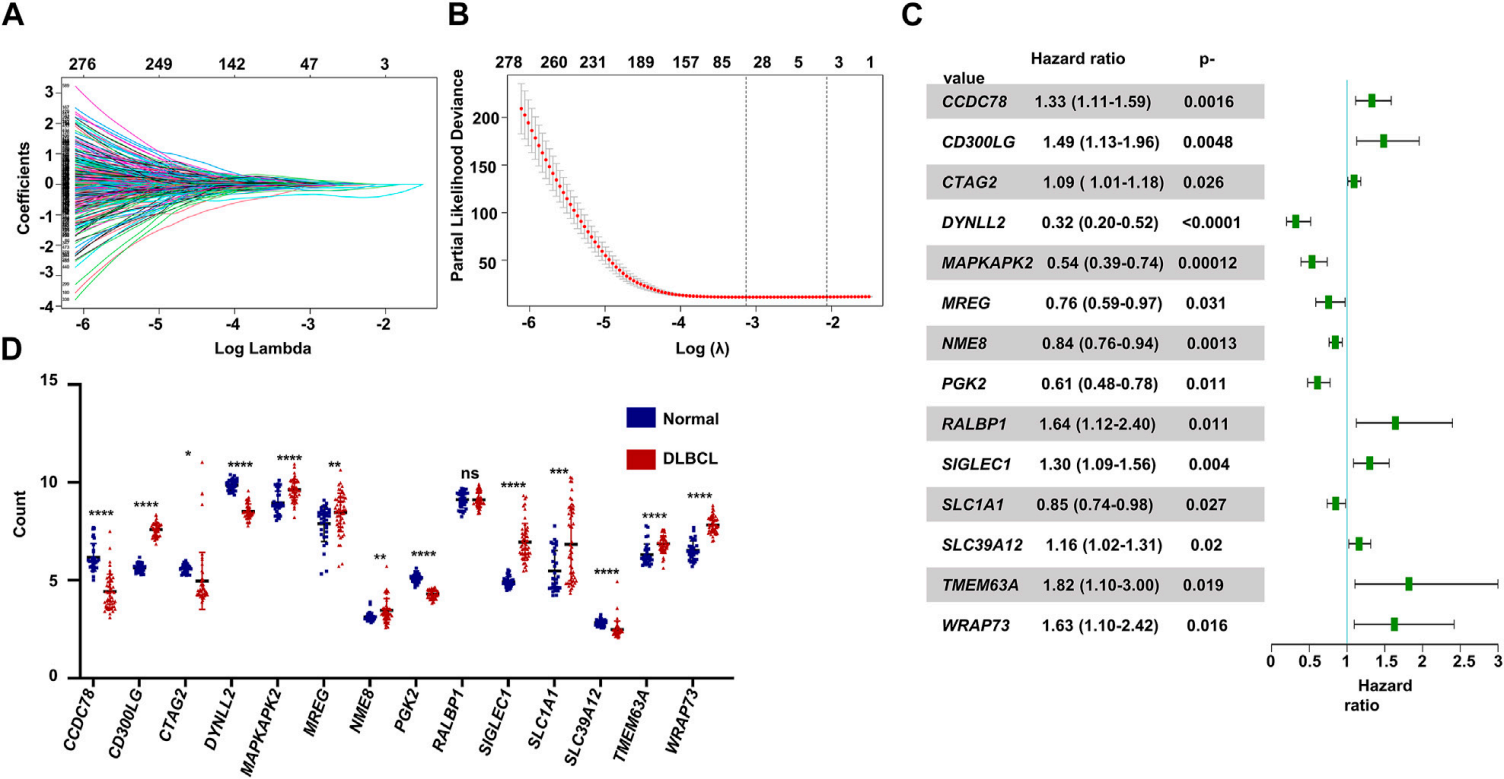
Construction of the prognostic gene signature. **(A)** LASSO regression analysis of the 596 prognosis-related genes. **(B)** Penalty plot for the LASSO regression analysis. **(C)** Forest plots of the multivariate Cox regression analyses of the 14 genes significantly associated with OS. **(D)** Expression levels of the 14 genes comparing normal B cells and DLBCL B cells. *p* < .05: *, *p* < .01: **, *p* < .001: ***, and *p* < .0001 ****.

The flowchart shown in Figure 1 illustrates our study process. First, we selected 1474 MAGs from the GSEA database and conducted univariate Cox regression analysis in the training dataset GSE10846. Next, we extracted 596 genes and performed least absolute shrinkage and selection (Lasso) penalized Cox regression analysis to screen the crucial prognostic genes based on the GSE10846 dataset. We calculated the coefficient values at different levels of penalty (Figure 2A). Next, we identified the optimal lambda (*λ*) value, and two best-fit values (lambda.min and lambda.1se) were determined by minimizing the mean-square error, thus establishing the Lasso models (Figure 2B). A total of 53 MAGs that correlated with OS were selected. Finally, we carried out multivariate Cox regression analyses and selected 14 potential genes. It was revealed that *CCDC78, CD300LG, CTAG2, DYNLL2, MAPKAPK2, MREG, NME8, PGK2, RALBP1, SIGLEC1, SLC1A1, SLC39A12, TMEM63A,* and *WRAP73* were significantly associated with the OS rate of DLBCL patients (Figure 2C and Supplementary Figures S1A–S1N). Furthermore, we explored the expression of the candidate genes mentioned previously in normal cells and DLBCL samples. Higher expression of *CD300LG, SIGLEC1, SLC39A12, TMEM63A,* and *WRAP73* was observed in DLBCL samples compared to normal controls. Meanwhile, lower expression of *DYNLL2, MAPKAPK2, MREG, NME8, PGK2,* and *SLC1A1* was detected in DLBCL samples (Figure 2D).

### 3.2 Relationship between candidate genes and survival

The functions of the selected genes listed in Table 2 indicate that most genes are associated with microtubules and are promising for use in prediction of the prognosis of DLBCL patients. We probed the independent influence of 14 MAGs on the OS of DLBCL patients and constructed the risk score model in light of the forum risk score = ∑ βi * Xi, where Xi is the gene expression level and βi is the regression coefficient.

**TABLE 2:** Functions of genes in the prognostic signature.

Gene	Function summary	Risk coefficient
*CCDC78*	Component of the deuterosome, a structure that promotes de novo centriole amplification in multiciliated cells	0.284559
*CD300LG*	May mediate L-selectin-dependent lymphocyte rollings	0.395619
*CTAG2*	encodes an autoimmunogenic tumor antigen that belongs to the ESO/LAGE family of cancer-testis antigens	0.089041
*DYNLL2*	Acts as one of several non-catalytic accessory components of the cytoplasmic dynein 1 complex	−1.137873
*MAPKAPK2*	Stress-activated serine/threonine-protein kinase involved in cytokine production, endocytosis, reorganization of the cytoskeleton, cell migration, cell cycle control. chromatin remodeling. DNA damage response and transcriptional regulation	−0.623684
*MREG*	Probably functions as cargo-recognition protein that couples cytoplasmic vesicles to the transport machinery	−0.279844
*NME8*	robably required during the final stages of sperm tail maturation in the testis and/or epididymis	−0.168762
*PGK2*	Essential for sperm motility and male fertility	−0.496461
*RALBP1*	Multifunctional protein that functions as a downstream effector of RALA and RALB	0.494892
*SIGLECI*	Acts as an endocytic receptor mediating clathrin dependent endocytosis.	0.262803
*SLC1A1*	Sodium-dependent, high-affinity amino acid transporter that mediates the uptake of L-glutamate and also L-aspartate and D-aspartate	-0.162554
*SLC39Al2*	Acts as a zinc-influx transporter	0.148126
*TMEM63A*	Acts as an osmosensitive calcium-permeable cation channel	0.598811
*WRAP73*	The SSX2IP:WRAP73 complex is proposed to act as regulator of spindle anchoring at the mitotic centrosome	0.487047

### 3.3 Construction and validation of the 14 MAGs risk score model

**FIGURE 3:**
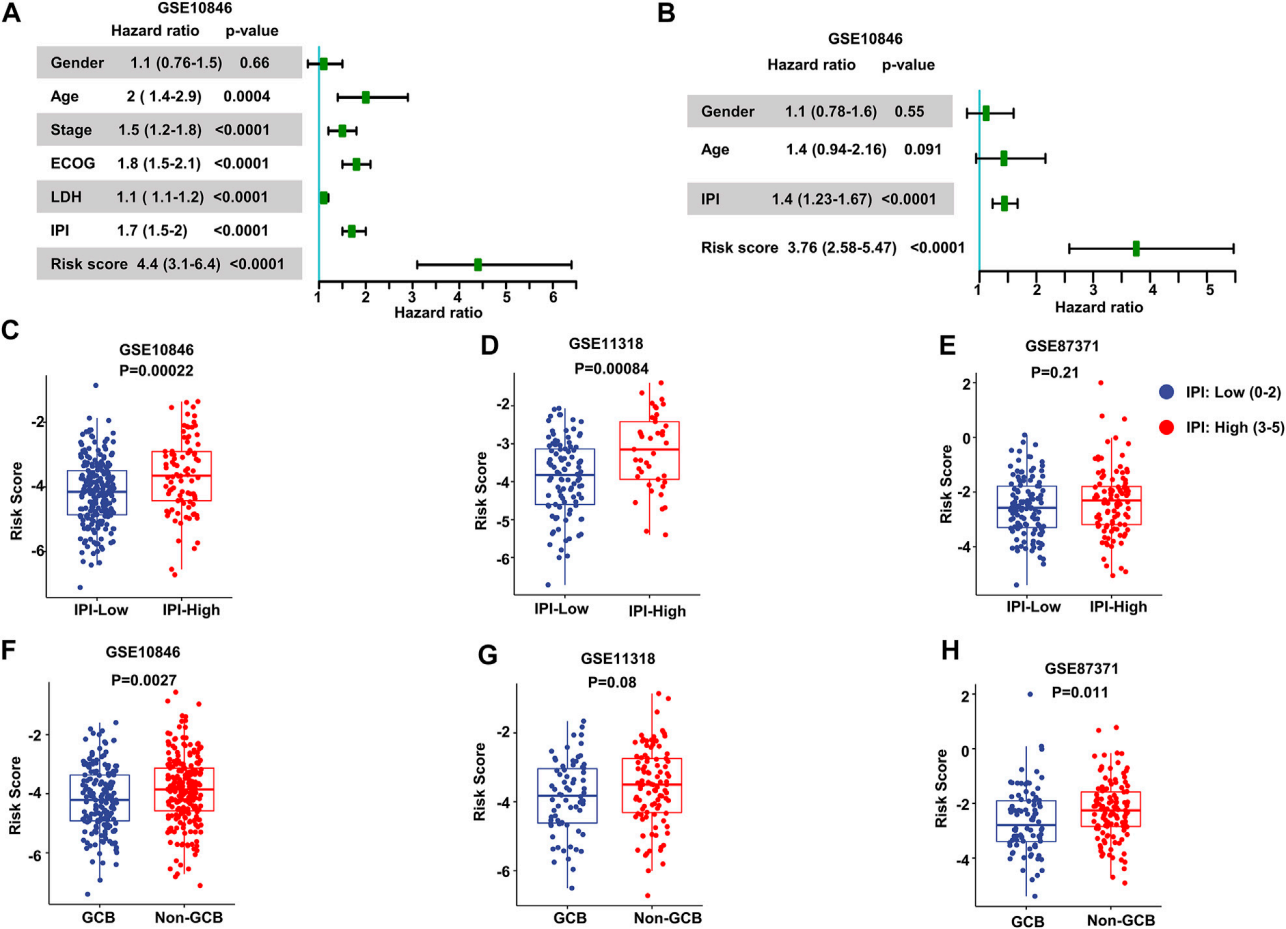
Validation of the prognostic model. **(A, B)** Forest plots of the univariate Cox regression analyses **(A)** and multivariate Cox regression analyses **(B)** of the prognostic risk score and clinical parameters in the training dataset GSE10846. **(C–E)** Box plots of the risk score in IPI-low and IPI-high groups in the training and validation datasets. **(F–H)** Box plots of the risk score comparing GCB and non-GCB subgroups. Training dataset: GSE10846; validation datasets: GSE11318 and GSE87371; IPI-low: 0–2, IPI-high: 3–5.

We performed univariate Cox regression and multivariate Cox regression analyses using the risk score and clinical parameters. These analyses showed that the IPI score and our MAG risk score were statistically associated with OS in the training and validation cohorts (Figures 3A,B and Supplementary Figures S2A–S2D). Subsequently, we performed subgroup analysis based on IPI scores and illustrated that MAG risk score was higher in subgroups with high IPI scores (3–5) in the GSE10846, GSE11318, and GSE87371 datasets (Figures 3C–E and Supplementary Figures S3A–S3C). Similarly, we performed subgroup analysis comparing germinal center B-cell (GCB) and non-GCB groups. The MAG risk score was higher in non-GCB groups, which suggested a worse prognosis for the subgroup of non-GCB patients (Figures 3F–H). We then compared age and stage in different risk score groups. A higher percentage of patients > 60 years of age and with stage 4 DLBCL were distributed in the high-risk group than in the low-risk group (Supplementary Figures S3D–S3I). Next, we compared the MAG model with the revised IPI (R-IPI), which used IPI to divide the patients into subgroups with zero risk factors, 1–2 risk factors, and 3–5 risk factors. The results showed that a higher proportion of patients with R-IPI of 3–5 belonged to the high-risk group (Supplementary Figures S4A–S4C). Lastly, we used the clinical parameters according to the National Comprehensive Cancer Network International Prognostic Index (NCCN-IPI) to validate the MAG model. Patients were divided into subgroups by age: ≤40, 41–60, 61–75, and >75 years, and then divided based on stage into subgroups of stage 1–2 and stage 3–4. We found that the high-risk group contained a higher percentage of patients aged 61–75 and > 75 years (Supplementary Figures S4D–S4F). In addition, patients with DLBCL of stage 3–4 had a higher risk score (Supplementary Figures S4G, S4H). These data suggest that our MAG model is consistent with clinical assessment methods such as GCB/non-GCB, IPI, R-IPI, and NCCN-IPI.

**FIGURE 4:**
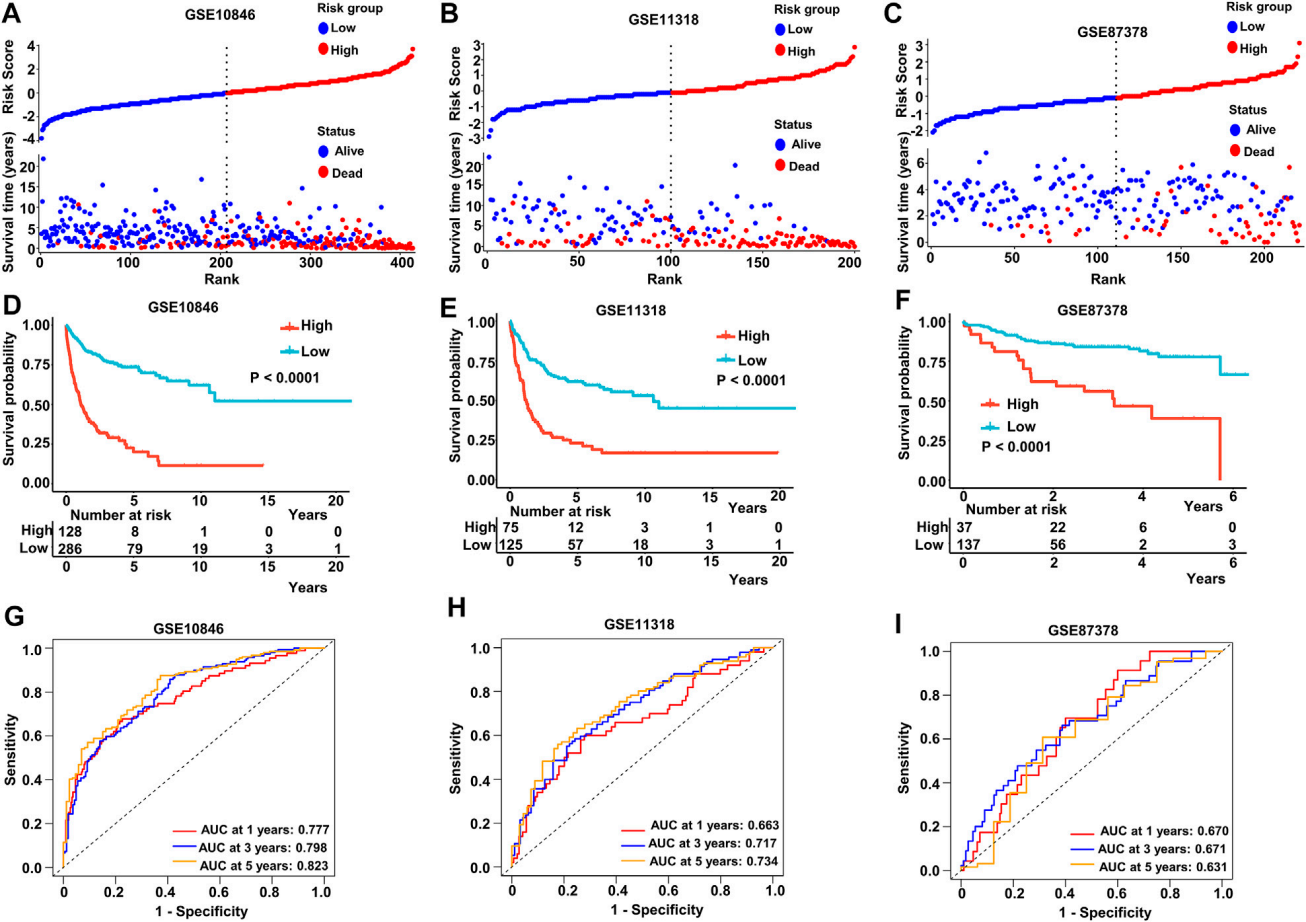
Predictive capacity of the prognostic gene signature. **(A–C)** Plot of the risk score, survival time, and status of patients in training and validation cohorts. **(D–F)** Kaplan–Meier curves of OS in the high-risk group and low-risk group in the training and validation sets. **(G–I)** ROC curves with calculated AUC of the training and validation datasets. Training dataset: GSE10846; validation datasets: GSE11318 and GSE87371.

We stratified patients into high- and low-risk groups based on the median risk score from the aforementioned multivariate Cox regression analysis. The results of our study demonstrated increased survival among patients in low-risk score groups. Conversely, increased numbers of fatalities were observed in the high-risk group in both the training and validating datasets (Figures 4A–C). After K-M analysis and comparing OS in the different datasets, a poorer outcome was noted in the high-risk group (*p* < .0001 in GSE10846, GSE11318, and GSE87378) (Figures 4D–F). To evaluate the accuracy of our newly constructed 14 MAG prognosis prediction model, we conducted a time-dependent ROC analysis in which the AUC at 1 year (.777), 3 years (.798), and 5 years (.823) in GSE10846 was analyzed (Figure 4G). Similarly, the AUC at 1, 3, and 5 years exhibited an excellent capacity to predict prognostic outcomes in GSE11318 and GSE87378 (Figures 4H, I). Consistent results and exceptional validation were observed in GSE31312 (Supplementary Figures S5A–S5C).

**FIGURE 5:**
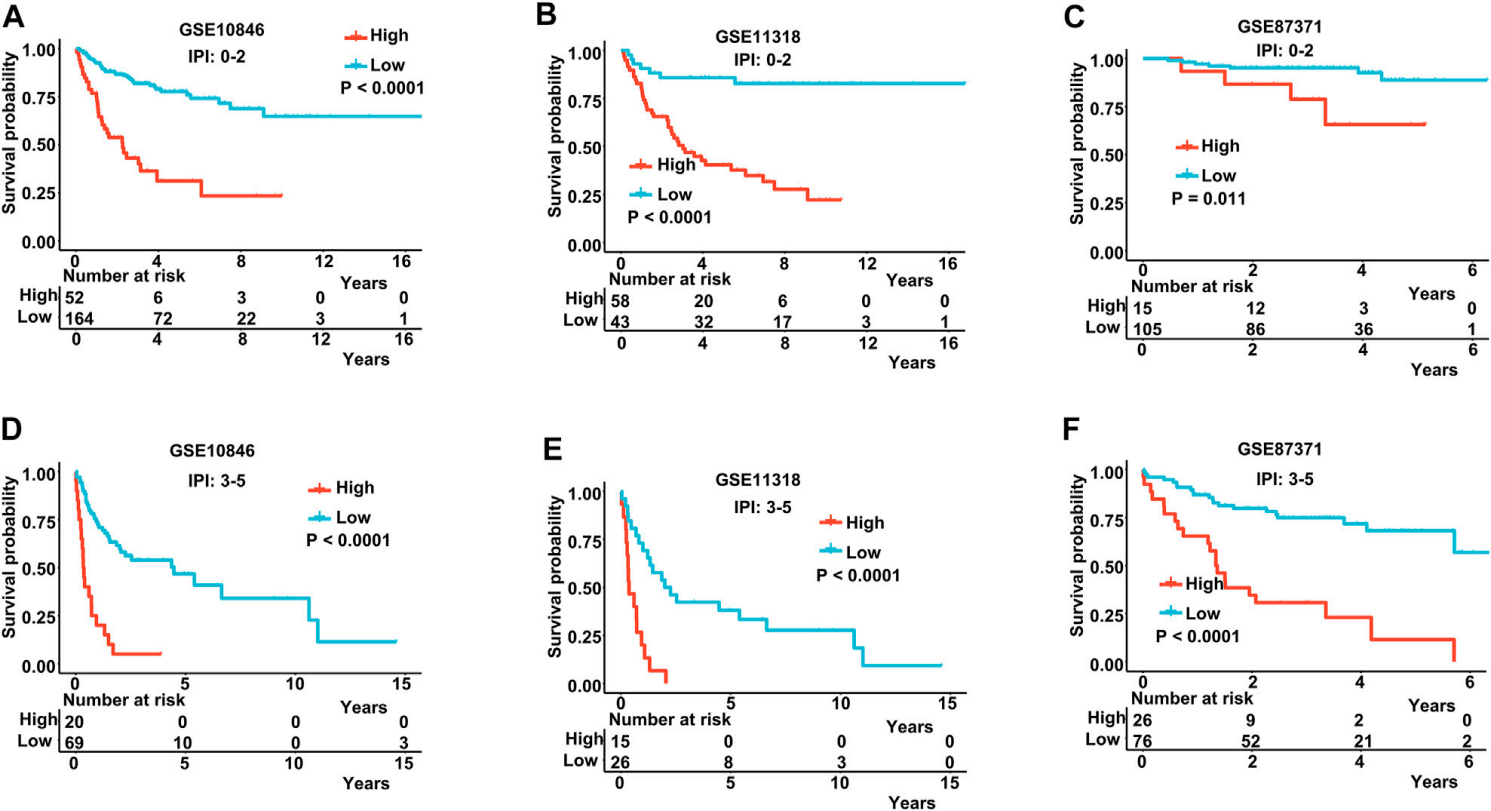
K-M curves in low-IPI score and high-IPI score subgroups. **(A–C)** K-M curves in low-IPI score groups in the training and validation datasets. **(D–F)** K-M curves in high-IPI score groups in the training and validation datasets. Training dataset: GSE10846; validation datasets: GSE11318 and GSE87371; IPI-low: 0–2, IPI-high: 3–5.

**FIGURE 6:**
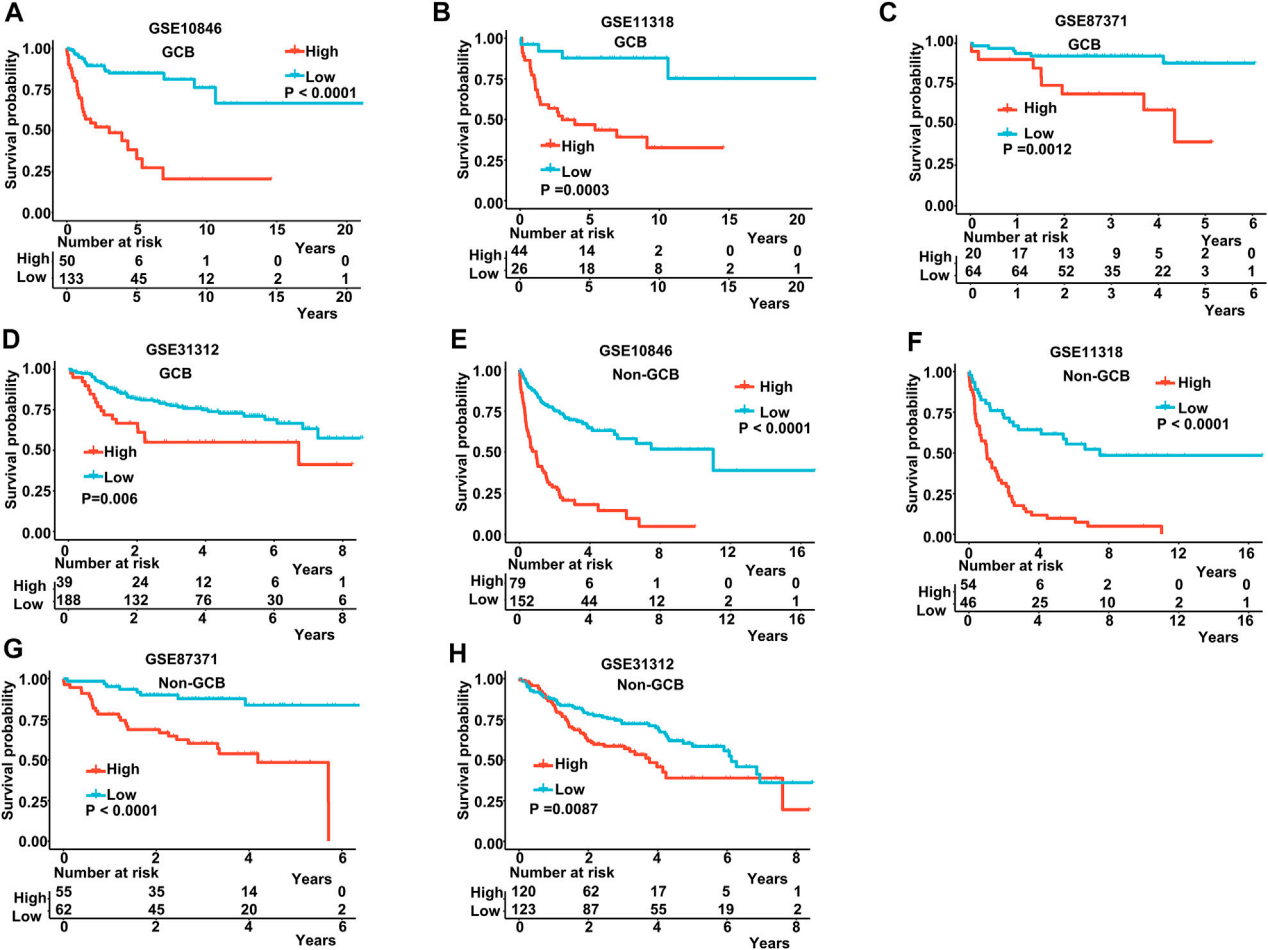
K-M curves in the GCB and non-GCB subgroups. **(A–D)** K-M curves in the GCB subgroups in the training and validation datasets. **(E–H)** K-M curves in the non-GCB subgroups in the training and validation datasets. Training dataset: GSE10846; validation datasets: GSE11318, GSE87371, and GSE11312.

The IPI score could not accurately distinguish the risk of individual DLBCL patients, so we tested our prognostic model in the high IPI score (3–5) and low IPI score (0–2) subgroups. K-M curves suggested an excellent prognostic value in different IPI score subgroups in both training and validating datasets (Figures 5A–F). Our risk score model also displayed good survival prediction in both GCB and non-GCB subgroups in different datasets (Figures 6A–H). Furthermore, we analyzed the prediction of the MAGs at 1, 3, and 5 years. The K-M curves markedly distinguished between low-risk and high-risk patients (Supplementary Figures S5D–S5L). These results suggest that the prognostic signature can predict the prognosis of most DLBCL patients.

### 3.4 Establishment of a nomogram for prognosis prediction

**FIGURE 7:**
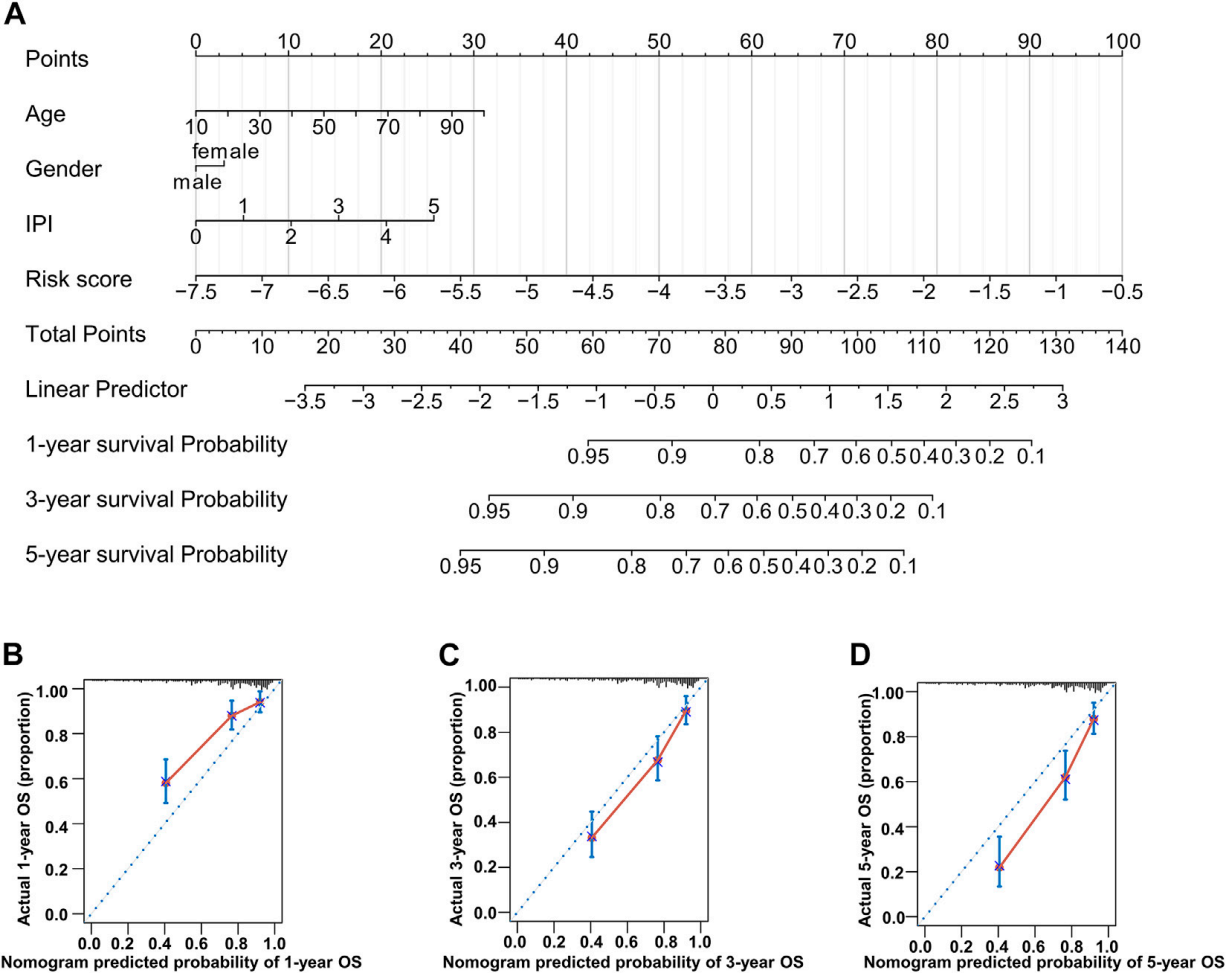
Nomogram consisting of age, gender, IPI score, and risk score. **(A)** Prognostic nomogram predicting OS at 1, 3, and 5 years in the training dataset. **(B–D)** Calibration curves for internal validation of the nomogram for OS at 1, 3, and 5 years.

To build a novel nomogram that provided more precise prognosis, we combined 14 MAG risk scores with the vital clinical factors of age, gender, and IPI scores (Figure 7A). The calibration curves of 1 year, 3 years, and 5 years were all very close to the ideal lines, indicating the powerful predictive capacity of this nomogram (Figures 7B–D).

### 3.5 Analysis of associated pathways

**FIGURE 8:**
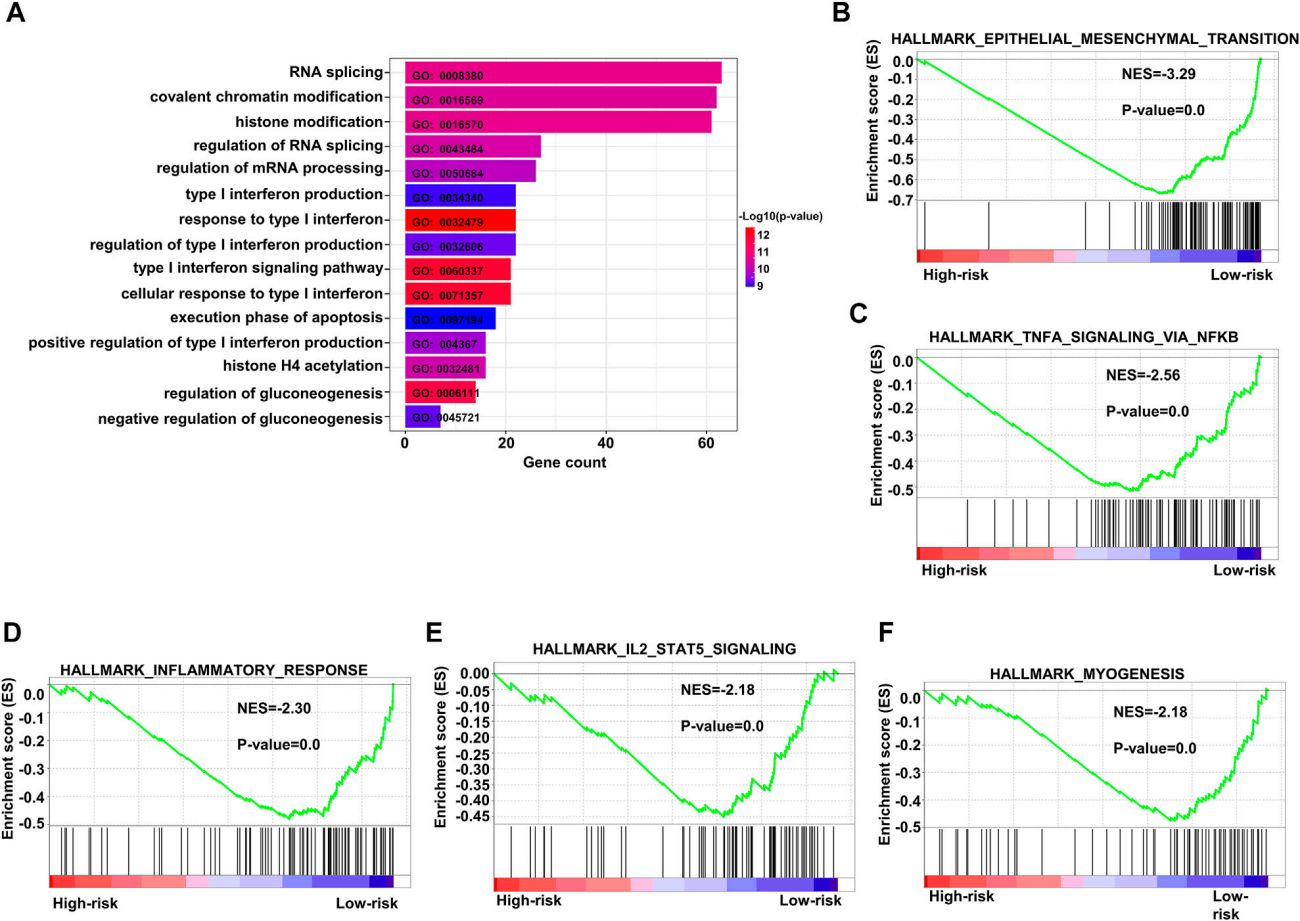
Enriched pathways in the high-risk group. **(A)** Bar plots of GO upregulated pathways in the high-risk score subgroup in the training dataset GSE10846. **(B–F)** Top five enriched pathways as indicated by GSEA of differentially expressed genes when comparing high-risk and low-risk groups in the training dataset. GO: gene ontology.

We performed GO analysis of upregulated genes in the population with high-risk scores and found that the enriched genes were mainly involved in RNA splicing, covalent chromatin modification, and histone modification (Figure 8A). We also conducted GSEA, and the results implied that the gene pathways enriched in patients with high-risk scores were most commonly related to upregulation of epithelial mesenchymal transition, inflammatory response, and myogenesis. In addition, enrichment of other crucial pathways involving NFKβ-TNFα and IL2-STAT5 signaling was also associated with high-risk patients (Figures 8B–F).

### 3.6 Drug sensitivity in different risk groups and immunohistochemical staining

**FIGURE 9:**
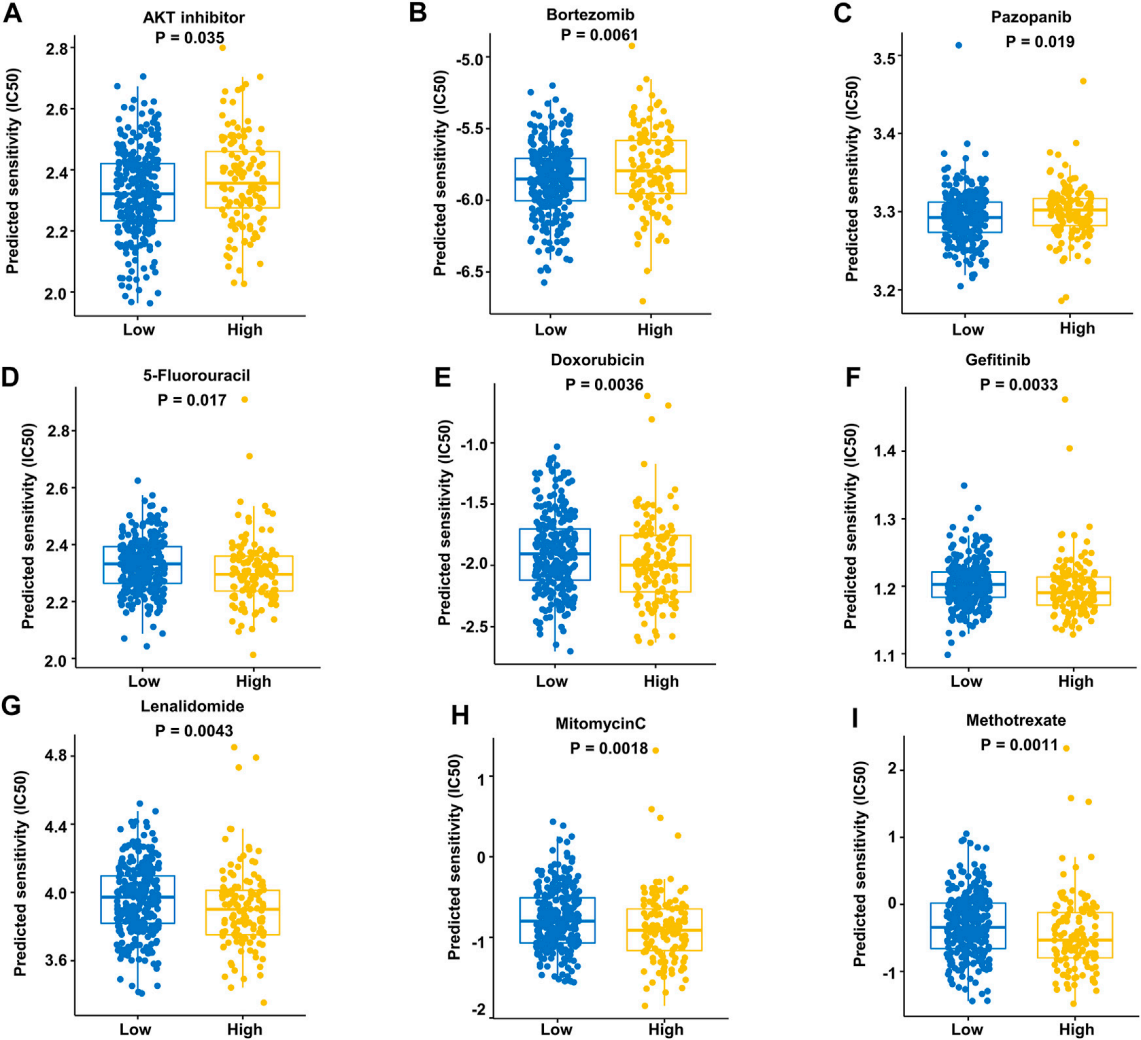
Prediction of responses to drugs. **(A–C)** Low-risk patients were more sensitive to these drugs in the training dataset. **(D–I)** High-risk patients were more sensitive to these drugs in the training dataset.

We assessed the drug sensitivity of high-risk and low-risk groups to conventional chemotherapeutic strategies. In our study, the low-risk group showed a significantly lower half-maximal inhibitory concentration (IC_50_) ratio of AKT inhibitors, bortezomib, and pazopanib (Figures 9A–C). In contrast, patients in the high-risk group were more sensitive to 5-fluorouracil, doxorubicin, gefitinib, lenalidomide, mitomycin C, and methotrexate than those in the low-risk group (Figures 9D–I). These data imply that AKT inhibitors, bortezomib, and pazopanib have promising effects in the low-risk groups, while 5-fluorouracil, doxorubicin, gefitinib, lenalidomide, mitomycin C, and methotrexate are recommended as treatment modalities in high-risk groups. These results indicate that it may be best to choose an inhibitor based on the different risk subgroups of each DLBCL patient.

**FIGURE 10:**
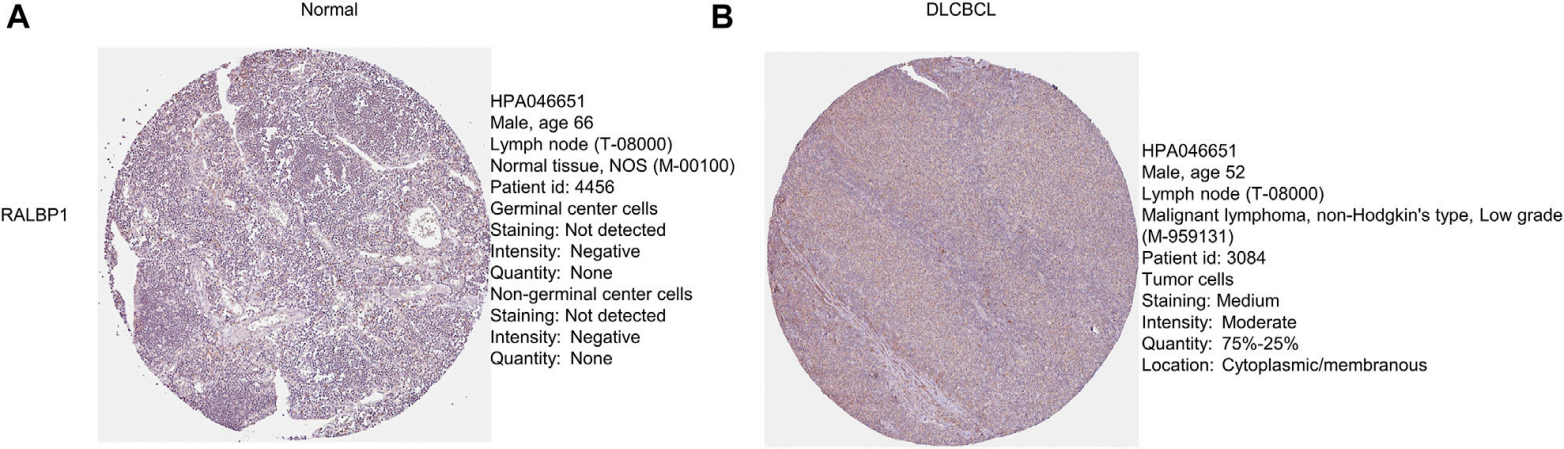
Protein levels of the prognostic genes. **(A, B)** Immunohistochemical staining of RALBP1 and MAPKAPK2 from the Human Protein Atlas database.

The level of RALBP1 protein expressed in DLBCL tissue was further validated using the Human Protein Atlas database (Figures 10A, B).

### 3.7 Inhibition of cell proliferation by *TMEM63A* knockout

**FIGURE 11:**
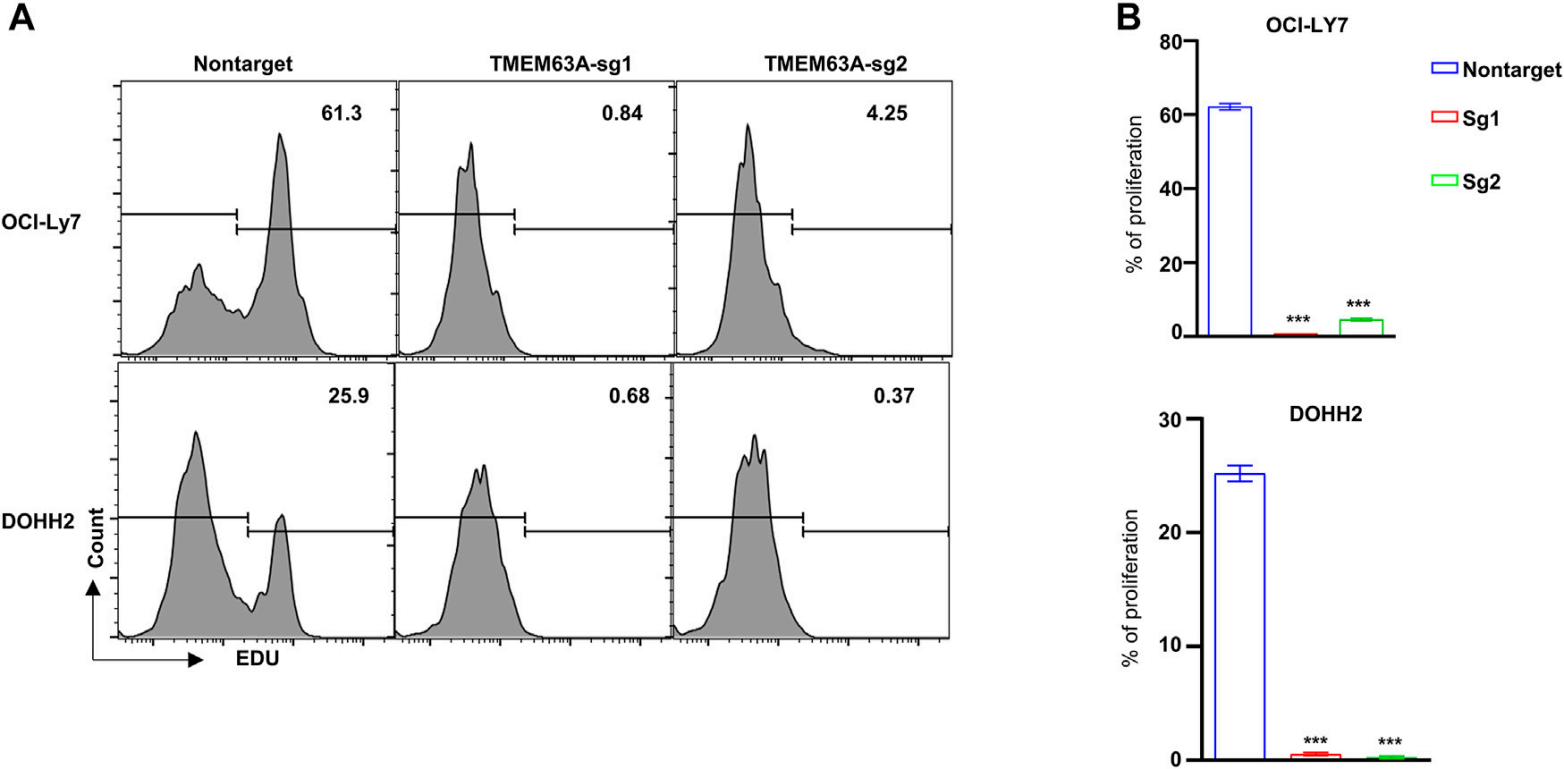
Knockout of the key prognostic gene *TMEM63A*. **(A)** Flow cytometric analysis of EdU staining after *TMEM63A* knockout. **(B)** Bar plots of cell proliferation.

To understand the function of *TMEM63A* in DLBCL, we generated *TMEM63A* knockout strains of the DLBCL cell lines OCI-LY7 and DOHH2 using CRISPR-CAS9-mediated sgRNA. Next, we analyzed the proliferating cells that showed growth inhibition following *TMEM63A* knockout using EdU staining (Figure 11A). Bar plots of the data showed a significant difference in proliferation of the control cell lines compared to the knockout cells (Figure 11B). These data indicate promise in targeting *TMEM63A* in DLBCL therapy.

## 4 Discussion

DLBCL is an aggressive lymphoma that damages normal lymph nodes and manifests extensive heterogeneity. Therefore, risk stratification and prognosis evaluations for DLBCL remain challenging for clinicians. The three currently available and universally acknowledged scoring systems in DLBCL (IPI, revised-IPI, and NCCN-IPI classifications) fail to identify high-risk patients with long-term OS below 50%. In our study, we analyzed the relationship between MAGs and the prognosis of patients with DLBCL in the GEO database and established a MAG prognostic model containing 14 genes (*CCDC78, CD300LG, CTAG2, DYNLL2, MAPKAPK2, MREG, NME8, PGK2, RALBP1, SIGLEC1, SLC1A1, SLC39A12, TMEM63A,* and *WRAP73*) that showed high predictive accuracy.

Microtubules are hollow tubes that radiate from the microtubule-organizing center situated at the centrosome in the cytoplasm of interphase eukaryotic cells. They are core cytoskeletal structures involved in material transport and cell proliferation that show promising capacity to reflect the prognosis of cancer patients (Osswald et al., 2016). However, the impact of microtubule function on prognostic assessment in DLBCL cases remains unclear. Our study demonstrated the use of MAGs to construct the first prognosis prediction model in DLBCL, which is capable of precisely evaluating prognosis for DLBCL patients.

Dynein light chain LC8-type 2(*DYNLL2*) is involved in cytoskeletal motor activity and protein binding, which plays an important role in the tumor microenvironment (Rapali et al., 2011). There is a negative correlation between *DYNLL2* expression and macrophage M0, the most abundant cells in stage N1 colorectal tumors (Ge et al., 2019). However, the correlation between DYNLL2 and hematolymphoid neoplasm has yet to be elucidated. Mitogen-activated protein kinase-activated protein kinase 2 (*MAPKAPK2* or *MK2*) is the downstream substrate in the p38MAPK pathway and induces post-translational regulation of cytokines (Soni et al., 2019). *MAPKAPK2* has been confirmed as the core regulator of RNA-binding proteins and has the ability to sustain regulation stability and inhibition of tumor progression (Suarez-Lopez et al., 2018). Similarly, melanoregulin (*MREG*) downregulates the phosphatidylinositol 3 kinase (PI3K)/Akt-mTOR signaling pathway and simultaneously inhibits the invasion and proliferation of cancer cells (Meng et al., 2017). *NME8* is the first metastasis suppressor protein found to be capable of suppressing metastasis of cancer cells without affecting primary tumor growth (Puts et al., 2018). Phosphoglycerate kinase 2 (*PGK2*) impacts the replication and repair of DNA in mammalian nuclei, and its expression is regulated *via* oxygen tension. It is a crucial enzyme in the glycolysis pathway, catalyzing glycerol-1,3-diphosphate into 3-phosphoglycerate conversion and ATP production (Wu et al., 2020). *SLC1A1* has been reported as a potential therapeutic target of natural killer T-cell lymphoma, and it contributes to the favorable prognosis of asparaginase-based anti-metabolic treatment (Xiong et al., 2021). Our research suggests that the aforementioned genes are protective factors in DLBCL.

*CCDC78* is correlated with poorer survival when using a prediction scoring model in colon cancer, but its role in other cancers is unclear. *CCDC78* has been found to interact with *PVT1*, which encodes lncRNA and maps to chromosome 8q24 (Guttman et al., 2009). Likewise, the oncogene *MYC,* a comprehensively acknowledged risk biomarker in DLBCL, has been mapped to 8q24. *MYC* was also found to co-amplify with *PVT1* in several cancer cell lines. *CD300LG* has a functional dependency associated with *WT1*, and upregulation and hypermethylation of *WT1* have been associated with poor prognosis (Ren et al., 2021). *CD300LG* also has a wide variety of immunological functions (Borrego, 2013). Immuno-proteomic screening demonstrated that elevated titers of auto-antibodies to the cancer-testis antigens (*CTAG2*) are correlated with diverse cancer types and suggest decreased differentiation in cancer cells (Kaaks et al., 2018). *RalBP1* acts as an important mediator of cancer cell migration (Lim et al., 2006). Wu et al. (2010) showed that *RalBP1* depletion inhibits cancer cell growth and metastasis *in vivo*. High expression of *SIGLEC1* was significantly associated with shorter DSS in an exploration of breast tumor-associated macrophage (TAM) markers. *CCL8* is chemotactic for monocytes and forms a positive regulatory loop between cancer cells and TAMs *via* CSF1 and TNF-α, which upregulates *SIGLEC1* (Lim et al., 2006). The solute carrier (SLC) groups of membrane transport proteins provide novel targets for therapeutic strategies in different types of malignant solid tumors, such as esophageal carcinoma, lung cancer, and pancreatic cancer (Cui et al., 2015; Wu et al., 2017). Significantly increased expression of *SLC39A12* has been associated with worse OS, especially for patients with positive lymph node metastasis (Liu et al., 2020). The transmembrane protein 63A (*TMEM63A*) has been reported to be a novel oncogene that promotes cell proliferation, migration, and invasion (Zhang et al., 2022). Xenograft tumor growth and lung metastasis were also observed *in vivo*. *WRAP73* encodes a member of the WD repeat protein family, which is implicated in osteoblast differentiation and osteogenesis *in vivo*. It has been proposed that *WRAP73* participates in the development of osteoporosis through regulation of bone remodeling. However, the role of *WRAP73* in DLBCL remains unknown. Given the results of our study, these genes can predict the prognosis of patients with DLBCL, and they show promise as novel therapeutic targets.

There were some limitations in our study. Further preclinical experiments are required to validate our predictive model and extend its capacity to inform clinical assessment. Nevertheless, through our study, we elucidated the association between MAGs and the prognosis of patients with DLBCL. The results of our study also suggest potential therapeutic targets and provide novel insights into the management of DLBCL.

## 5 Conclusion

In summary, we constructed a reliable MAG signature capable of predicting survival and showing remarkable prognostic performance. We also discussed distinctive therapeutic patterns in high- and low-risk cohorts. In addition to being prognostic biomarkers for DLBCL, the candidate genes in our predictive model are promising potential therapeutic targets.

## Data Availability

The original contributions presented in the study are included in the article/Supplementary Material; further inquiries can be directed to the corresponding authors.
