# Corrigendum: Identification of microtube-associated biomarkers in diffuse large B-cell lymphoma and prognosis prediction

**DOI:** 10.3389/fgene.2023.1180076

**Published:** 2023-06-08

**Authors:** Wenqi Wu, Su Liu, Linyan Tian, Cheng Li, Yanan Jiang, Jinhuan Wang, Yangyang Lv, Jing Guo, Donghui Xing, Yixin Zhai, Huimeng Sun, Yuhang Li, Luying Zhang, Xiang He, Kaiping Luo, Hongjie Zhan, Zhigang Zhao

**Affiliations:** ^1^ Department of Hematology, Tianjin Medical University Cancer Institute and Hospital, National Clinical Research Center for Cancer, Key Laboratory of Cancer Prevention and Therapy, Tianjin’s Clinical Research Center for Cancer, Tianjin, China; ^2^ Department of Medical Oncology, Tianjin First Central Hospital, School of Medicine, Nankai University, Tianjin, China; ^3^ Department of Pharmacy, Shandong Cancer Hospital and Institute, Shandong First Medical University and Shandong Academy of Medical Sciences, Jinan, China; ^4^ Department of Gastroenterology, Tianjin Medical University Cancer Institute and Hospital, National Clinical Research Center for Cancer, Key Laboratory of Cancer Prevention and Therapy, Tianjin’s Clinical Research Center for Cancer, Tianjin, China

**Keywords:** DLBCL, microtube-associated genes, prognostic model, targeting therapy, TMEM63A

In the published article, there was an error in Affiliation(s) “3”. Instead of “Department of Gastroenterology, Tianjin Medical University Cancer Institute and Hospital, National Clinical Research Center for Cancer, Key Laboratory of Cancer Prevention and Therapy, Tianjin’s Clinical Research Center for Cancer, Jinan, China”, it should be “Department of Pharmacy, Shandong Cancer Hospital and Institute, Shandong First Medical University and Shandong Academy of Medical Sciences, Jinan, China.”

Also, for author “Hongjie Zhan”, the city in the affiliation is corrected from “Jinan” to “Tinajin” and its was renumbered to 4: “Department of Gastroenterology, Tianjin Medical University Cancer Institute and Hospital, National Clinical Research Center for Cancer, Key Laboratory of Cancer Prevention and Therapy, Tianjin’s Clinical Research Center for Cancer, Tianjin, China.”

The authors apologize for this error and state that this does not change the scientific conclusions of the article in any way. The original article has been updated.

